# Disruption of Retinoic Acid Receptor Alpha Reveals the Growth Promoter Face of Retinoic Acid

**DOI:** 10.1371/journal.pone.0000836

**Published:** 2007-09-05

**Authors:** Giulia Somenzi, Giusy Sala, Stefano Rossetti, MingQiang Ren, Riccardo Ghidoni, Nicoletta Sacchi

**Affiliations:** 1 Cancer Genetics Program, Department of Cancer Biology, Roswell Park Cancer Institute, Buffalo, New York, United States of America; 2 Laboratory of Biochemistry and Molecular Biology, San Paolo University Hospital, School of Medicine, University of Milan, Milan, Italy; Vanderbilt University, United States of America

## Abstract

**Background:**

Retinoic acid (RA), the bioactive derivative of Vitamin A, by epigenetically controlling transcription through the RA-receptors (RARs), exerts a potent antiproliferative effect on human cells. However, a number of studies show that RA can also promote cell survival and growth. In the course of one of our studies we observed that disruption of RA-receptor alpha, RARα, abrogates the RA-mediated growth-inhibitory effects and unmasks the growth-promoting face of RA (Ren et al., Mol. Cell. Biol., 2005, 25:10591). The objective of this study was to investigate whether RA can differentially govern cell growth, in the presence and absence of RARα, through differential regulation of the “rheostat” comprising ceramide (CER), the sphingolipid with growth-inhibitory activity, and sphingosine-1-phosphate (S1P), the sphingolipid with prosurvival activity.

**Methodology/Principal Findings:**

We found that functional inhibition of endogenous RARα in breast cancer cells by using either RARα specific antagonists or a dominant negative RARα mutant hampers on one hand the RA-induced upregulation of neutral sphingomyelinase (nSMase)-mediated CER synthesis, and on the other hand the RA-induced downregulation of sphingosine kinase 1, SK1, pivotal for S1P synthesis. In association with RA inability to regulate the sphingolipid rheostat, cells not only survive, but also grow more in response to RA both *in vitro* and *in vivo*. By combining genetic, pharmacological and biochemical approaches, we mechanistically demonstrated that RA-induced growth is, at least in part, due to non-RAR-mediated activation of the SK1-S1P signaling.

**Conclusions/Significance:**

In the presence of functional RARα, RA inhibits cell growth by concertedly, and inversely, modulating the CER and S1P synthetic pathways. In the absence of a functional RARα, RA–in a non-RAR-mediated fashion–promotes cell growth by activating the prosurvival S1P signaling. These two distinct, yet integrated processes apparently concur to the growth-promoter effects of RA.

## Introduction

Retinoic acid (RA), the bioactive derivative of dietary Vitamin A and beta-carotene, is a powerful signaling molecule controlling cell proliferation [1]. Normally, RA exerts an inhibitory action on cell proliferation *via* specialized transcription factors, the nuclear RA receptors, RARs [2]. Cells evolved an amazing apparatus to safely control the RA antiproliferative action. First, cells finely regulate the level of intracellular RA through a sophisticated metabolic/homeostatic process [3]; second, they use specialized cellular retinoic acid binding proteins (CRABPs) to chaperone RA from the cytoplasm directly onto the RARs in the nucleus [4]; third, they have evolved a two-tier RAR-regulated system to control the downstream transcription of genes in response to RA [5]. RA, after binding the nuclear receptor RARα, triggers the transcription of other downstream RARs, including the RA-receptor and tumor suppressor, *RARβ2* [6]. RARβ2 autoregulates its own transcription and, in turn, the transcription of a multitude of downstream RA-responsive target genes [7], [8].

Heterogeneous factors can lead to functional inhibition of RA-RARα signaling. These factors include non-random genetic mutations producing chimeric RARα receptors with dominant negative function, such as the leukemia-associated PML-RARα and PLZF-RARα [9 and references within], *RARα* epigenetic silencing in epithelial cancer cells [10]–[13], and a defective intracellular level of RA consequent to defects of the retinol/RA metabolism/homeostasis [4], [14]–[17].

According to several literature reports, RA and its dietary precursors can also promote, rather than inhibit, cell survival and growth [18]–[22]. In the course of a recent study, we observed that disruption of RARα signaling in RA-sensitive breast cells not only leads to RA-resistance, but unexpectedly unmasks the growth-promoter face of RA [13], [23]–[25].

Here we show that in RA-sensitive cells with a functional RARα signaling RA leads to growth inhibition consequent to the concerted upregulation of neutral sphingomyelinase (nSMase), one of the enzymes leading to the synthesis of the antiproliferative/propaptotic ceramide (CER), and downregulation of sphingosine kinase 1 (SK1), the enzyme leading to the synthesis of the prosurvival sphingosine-1-phosphate (S1P). In contrast, disruption of RARα signaling in the same cells results, in response to RA, into increased proliferation associated with both loss of concerted regulation of nSMase and SK1, and induction of intracellular S1P. Altogether our findings indicate that the presence of RARα is essential for the proper regulation of the sphingolipid rheostat by RA. In the absence of RARα, RA no longer executes its growth-inhibitory action through its canonical receptors, but activates the prosurvival SK1-S1P pathway through alternate non-RAR receptor(s).

## Results

### Cells with a functionally disrupted RARα signaling become both RA-resistant and susceptible to RA-induced cell growth *in vitro* and *in vivo*

**Figure 1:**
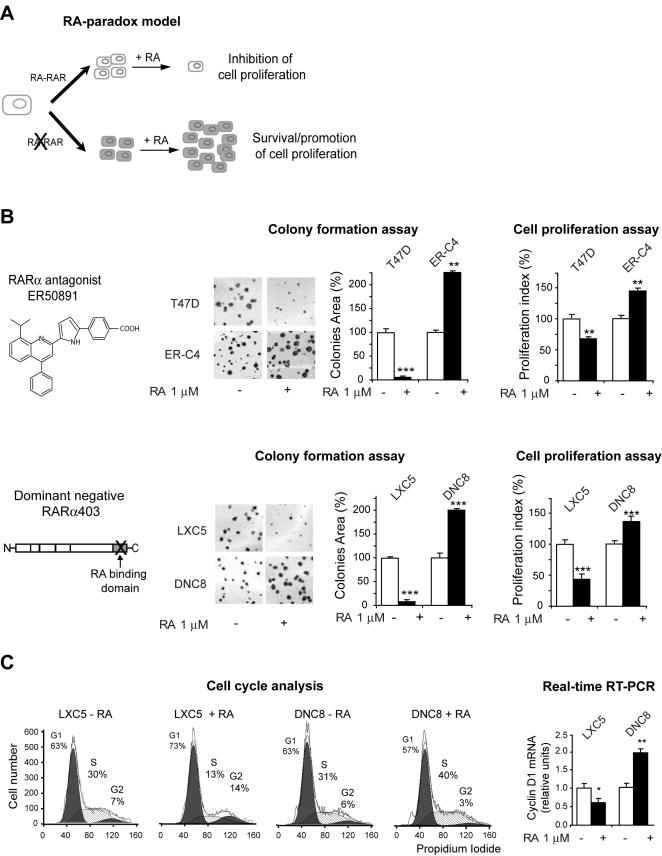
Cells with a functionally disrupted RARα signaling become both RA-resistant and susceptible to RA-induced cell growth. A) Differential response to RA according to the integrity of the RARα signaling. B) Both the ER-C4 clone, in which RA signaling was impaired by treatment with the RARα antagonist ER50891 (top), and the DNC8 clone, expressing the dominant negative RARα403 (bottom), were growth-stimulated by RA as shown by both colony formation assay and cell proliferation assay, while their cognate controls (T47D and LXC5, respectively) were growth inhibited. C) RA expedites the G1-S transition in DNC8 cells (left), and significantly induces cyclin D1 transcription (right).

By using different strategies to functionally inhibit RA-RARα signaling in RA-sensitive breast cancer cells (T47D), we found that–concomitant with heritable epigenetic gene silencing of the downstream RARβ2 receptor and tumor suppressor–cells not only survive, but also proliferate significantly more in response to RA ([13] and Fig. 1A). Specifically, we observed that T47D-derived clones, obtained by stably inhibiting the endogenous RARα signaling with either the RARα-specific antagonist ER50891 (here shown a prototypic clone, ER-C4) (Fig. 1B, top) or the dominant negative DN RARα403 mutant (here shown a prototypic clone, DNC8) (Fig.1B, bottom), were not only RA-resistant, but grew significantly more in the presence of RA (1 µM, 72 h) as shown by both colony formation assay and cell proliferation assay, while their respective controls, T47D in the case of ER-C4, and LXC5 in the case of DNC8, were growth-inhibited by RA. We will present hereafter only the results concerning the LXC5/DNC8 isogenic model, because the T47D/ER-C4 show an identical RA-response for the different parameters that we analyzed in this study.

The increased RA-induced growth is supported by the observation that cells in the presence of RA (1 µM, 72 h) transition more rapidly from the G1 to the S phase (Fig. 1C, left) in agreement with an increased transcription of the cyclin D1 gene, encoding a protein pivotal for the G1-S phase transition (Fig.1C, right).

**Figure 2:**
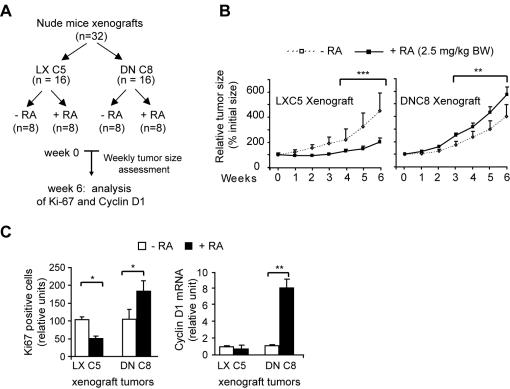
RA promotes tumor growth *in vivo*. A) LXC5 and DNC8 cells were xenografted in nude mice, which were treated with RA according to the experimental scheme. B) Weekly assessment of tumor size over six weeks showed that DNC8 xenograft tumors of mice that received RA treatment grew significantly more than tumors of mice that did not received RA treatment (right). LXC5 xenograft tumors underwent growth inhibition in response to RA (left). C) RA significantly promoted cell proliferation as demonstrated by a significantly higher number of Ki67-positive cells (left) and increased cyclin D1 transcription (right) in RA-treated DNC8 cells.

RA, and its dietary precursor retinol, can apparently promote DNC8 cell growth *in vivo*. DNC8 cells xenografted subcutaneously, and bilaterally, in the dorsal flank of female nude mice (see experimental scheme, Fig. 2A), were clearly growth-promoted by chronic RA treatment (2.5 mg/kg) delivered by daily intraperitoneal injection. Weekly assessment of tumor size showed that RA clearly promoted the growth of DNC8 xenograft tumors up to the sixth week (Fig. 2B, right); thereafter tumors stop growing (data not shown). In contrast, the same RA treatment induced growth inhibition of the control LXC5 xenograft tumors (Fig. 2B, left). Immunocytochemistry of DNC8 tumor sections after six-week RA-treatment showed a significantly (p<0.05) higher number of cells positive for the proliferation marker Ki67 (Fig. 2C, left). Significantly (p<0.01) higher was also the level of *cyclin D1* transcription (Fig. 2C, right).

Based on the overall *in vitro* and *in vivo* observations, we hypothesized that two distinct effects occur as a consequence of disruption of RARα function. The first effect is the abrogation of growth inhibition mediated by RA through RARα, and the second effect is a non-RARα-mediated stimulation of cell growth by RA itself. To identify these effects we focused our analysis on CER and S1P signaling, two sphingolipid signaling pathways exerting opposite action on cell growth.

### RA fails to induce CER synthesis in cells with functional RARα inhibition

**Figure 3:**
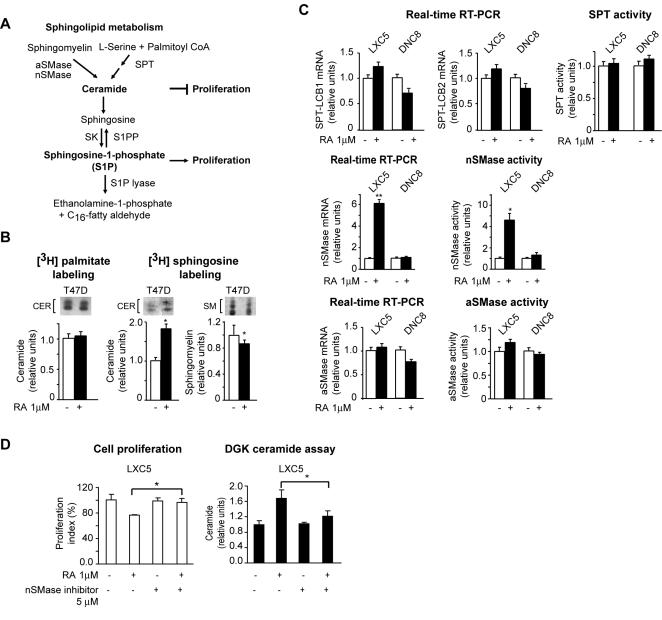
RA fails to induce nSMase-mediated CER synthesis in cells with functional RARα inhibition. A) Scheme showing the metabolic pathways leading to the synthesis of both CER and S1P, two bioactive sphingolipids known for exerting opposite effects on proliferation. B) [^3^H] palmitate or [^3^H] sphingosine labelling, in the presence and absence of RA, showing that RA induces CER accumulation through a sphingomyelinase pathway (fluorographic pattern of CER and sphingomyelin (SM), respectively, in the upper inserts). C) Quantitative analysis of transcription and activity for the different enzymes leading to CER synthesis in both LXC5 cells and DNC8 cells clearly indicates that RA induces CER through the nSMase pathway (middle) and not the SPT (top) and aSMase (bottom) pathways. D) The nSMase inhibitor GW4869 significantly counteracted both RA-induced growth inhibition (left) and CER accumulation (right).

The metabolism of both CER and S1P is tightly integrated (Fig. 3A). CER can be generated either as a result of sphingomyelin hydrolysis, catalyzed by either one of two sphingomyelinases, the neutral sphingomyelinase (nSMase) and the acid sphingomyelinase (aSMase), or by *de novo* synthesis as a result of condensation of L-serine and palmitoyl CoA catalyzed by serine palmitoyltransferase (SPT) (Fig. 3A). In preliminary cell labeling experiments of T47D cells with either [^3^H] sphingosine or [^3^H] palmitate, it was apparent that RA induces CER synthesis *via* sphingomyelin hydrolysis, and not *de novo* synthesis in cells with a functional RARα (Fig. 3B). Consistently, both the transcription level of the two SPT subunits genes, *LCB1* and *LCB2*, and the SPT activity did not vary significantly between LXC5 and DNC8 cells in response to RA (Fig. 3C, top). In contrast, both the transcription level and activity of one of the sphingomyelinases, nSMase (Fig. 3C middle), significantly (p<0.01) increased in response to RA in LXC5, but not in DNC8 cells. Conversely, the transcription and activity of aSMase remained unchanged (Fig. 3C bottom). Moreover, a specific nSMase inhibitor, GW4869 [26] (5 µM, 48 h) significantly (p<0.05) counteracted RA-induced growth inhibition (Fig. 3D, left) as well as RA-induced CER level in LXC5 cells (Fig. 3D, right).

**Figure 4:**
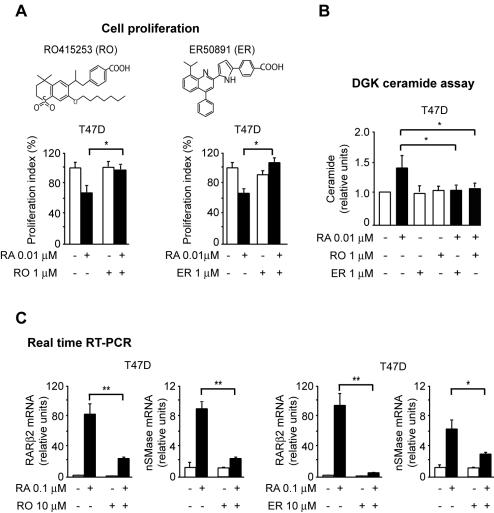
RARα antagonists counteract both RA-induced growth inhibition and nSMase-mediated CER synthesis. A) RO415253 (RO) (left) and ER50891 (ER) (right) rescued T47D cells from RA-induced growth-inhibition. B) Both RO and ER counteracted the RA-induced CER synthesis. C) Both RO and ER significantly counteracted the transcription of *nSMase* as well as the transcription of the control downstream RARα direct target *RARβ2*.

To validate independently whether nSMase-driven CER synthesis is under RARα regulation, we used two specific RARα antagonists RO415253 [27] and ER50891 [28]. Both antagonists effectively inhibit RA action at RARα, since they abrogate RA-induced transcriptional upregulation of RARβ2, a prototypic direct RARα-target (Fig. 4C). Treatment of T47D cells with a 100-fold concentration of either one of the RARα antagonists relative to RA for 72 h, counteracted both the RA-induced antiproliferative activity (Fig. 4A) and the RA-induced CER synthesis in T47D cells (Fig. 4B). Further, both RARα antagonists inhibited the RA-induced transcriptional upregulation of nSMase (Fig. 4C). These findings demonstrate a functional interference of both antagonists with RA-induced, nSMase-mediated CER synthesis.

Thus, by using two independent approaches, we clearly demonstrated that disruption of RARα function abrogates the nSMase-mediated synthesis of CER in response to RA.

### Fenretinide, a retinoid that works in an RAR-independent fashion, can induce CER in cells with functional RARα inhibition

**Figure 5:**
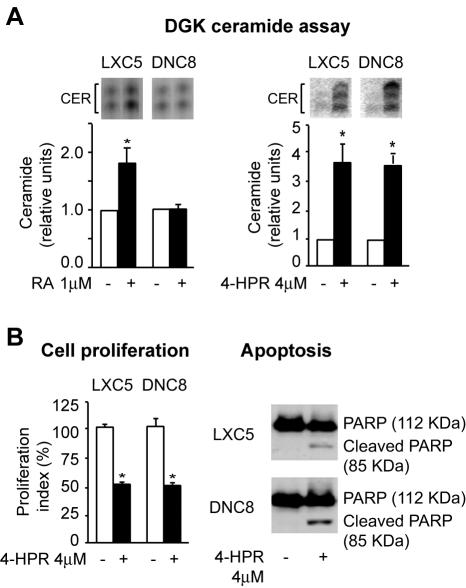
Fenretinide can induce CER also in cells without a functional RARα. A) DGK analysis of CER (autoradiography of CER species in the upper inserts) showing that RA induces CER only in cells with a functional RARα signaling (left), while fenretinide, a retinoid whose action is RAR-independent, can induce CER also in cells without a functional RARα signaling (right). B) Fenretinide-induced CER accumulation has an antiproliferative and proapoptotic effect, as shown by cell proliferation assay (left) and the presence of cleaved PARP (right), respectively.

Fenretinide (4-HPR) is a synthetic retinoid that was shown to be effective for prevention and treatment of breast cancer [29], [30]. Fenretinide was reported to induce CER accumulation in a non-RAR-dependent fashion [31], [32]. Consistently, DNC8 cells, while unable of nSMase-induced CER synthesis in response to RA (1 µM, 72 h) (Fig. 5A left), were capable of accumulating CER in response to fenretinide (4 µM, 72h) (Fig. 5A, right). Fenretinide-induced CER accumulation is paralleled both by a consistent antiproliferative (Fig. 5B, left) and proapoptotic effect (Fig. 5B, right).

These observations support the conclusion that the inability of RA to induce CER synthesis in cells with functional disruption of RARα is due to lack of upregulation of the specific nSMase-mediated CER synthetic pathway, and not to an overall failure of the entire CER synthetic apparatus.

### RA fails to downregulate both SK1 transcription and activity in cells with functional RARα inhibition

**Figure 6:**
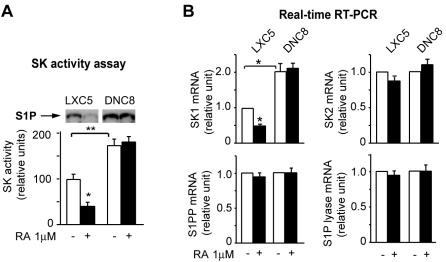
RA fails to downregulate both *SK1* transcription and activity in cells with functional RARα inhibition. A) Downregulation of SK1 activity (S1P spots in the upper insert) in response to RA occurs in LXC5 cells but not DNC8 cells. B) *SK1* transcription (but not the transcription of *SK2, S1PP, S1P lyase*) is downregulated in response to RA in LXC5 cells but not in DNC8 cells.

The metabolism of the antiproliferative CER is intrinsically linked to the metabolism of the prosurvival S1P effector (Fig. 3A). For this reason, we measured the SK activity in LXC5 and DNC8 cells both at baseline (in the absence of RA) and in the presence of RA. Apparently, LXC5 cells have a significantly (p<0.01) lower level of SK1 activity than DNC8 cells already at baseline (Fig. 6A). Further, RA (1 µM, 72 h) can induce a significant downregulation (p<0.05) of SK1 activity in LXC5 but not in DNC8 cells (Fig. 6A, upper inserts). The SK1 activity pattern in LXC5 and DNC8, at baseline and after RA-treatment, mirrors the transcription pattern of the *SK1* gene, one of the two *SK* genes (Fig. 6B, top left). The transcription of sphingosine kinase 2 (*SK2*), sphingosine-1-phosphate lyase (*S1P lyase*), and sphingosine-1-phosphate phosphatase (*S1PP*) is not significantly different in LXC5 and DNC8 cells both at baseline and after RA-treatment (Fig. 6B, top right, and bottom).

Apparently, only in cells with a functional RARα, RA transcriptionally regulates in an opposite fashion the metabolic pathways leading to either CER or S1P synthesis, by upregulating on one hand *nSMase* transcription and by downregulating on the other hand *SK1* transcription, thus synergistically inhibiting cell proliferation.

### Evidence that RA fails to regulate in an opposite fashion CER synthesis and SK activity in cells lacking endogenous RARα

**Figure 7:**
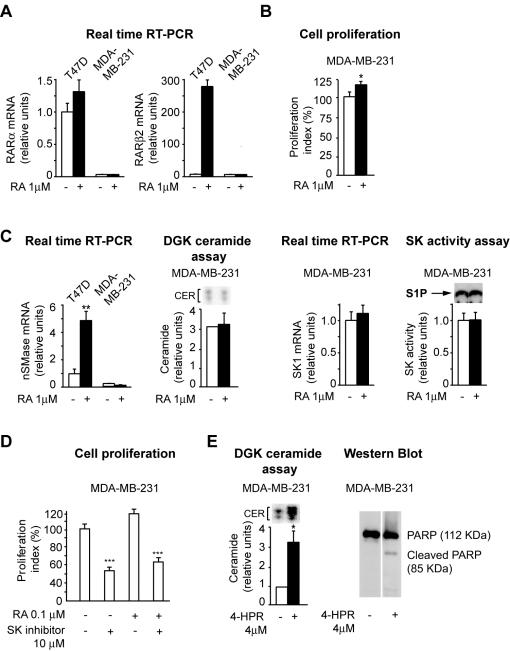
Evidence that RA fails to regulate in an opposite fashion CER synthesis and SK activity in cells lacking endogenous RARα. A) Lack of endogenous RARα signaling (left) is demonstrated by lack of RA-induced transcription of the RARα target *RARβ2* (right). B) MDA-MB-231 are modestly, but significantly, growth-promoted by RA. C) RA fails to induce *nSMase* transcription (left), CER synthesis (middle), *SK1* transcription downregulation (middle), and decrease of SK activity (right) in MDA-MB-231 cells. D) RA-induced MDA-MB-231 proliferation is significantly decreased by treatment with a SK inhibitor. E) Fenretinide can effectively induce both CER synthesis (left) and apoptosis (right) in MDA-MB-231 cells.

Next, we searched for evidence that RA fails to regulate in an opposite fashion CER synthesis and SK activity also in cells that lack endogenous RARα function. For this reason, we chose a breast cancer cell line, MDA-MB-231, that does not express endogenous *RARα* (Fig 7A, left) and the other downstream RA-regulated RAR genes, including *RARβ2* (Fig. 7A, right). MDA-MB-231 are modestly, yet significantly (p<0.05) growth-promoted by RA (Fig. 7B). Interestingly, in these cells RA fails to: induce *nSMase* transcription (Fig. 7C, left), increase CER synthesis (Fig 7C, middle), and downregulate both SK transcription and activity (Fig. 7C, middle and right).

Treatment with the SK inhibitor 2-(p-hydroxyanilino)-4-(p-chlorophenyl) thiazole (2 µM, 72 h) led to significant inhibition (p<0.01) of MDA-MB-231 proliferation both in the absence and presence of RA (Fig. 7D), indicating that RA-promoted cell proliferation of MDA-MB-231 cells might be due, at least in part, to activation of the SK1-S1P signaling pathway. In addition, MDA-MB-231 can accumulate CER (Fig. 7E, left) and undergo apoptosis (Fig. 7E, right) in response to fenretinide, showing that other non-RAR-regulated ceramide synthetic pathways are still functional. Thus, in different cell contexts, both when we disrupted functional RARα (DNC8) or there is no endogenous RARα (MDA-MB-231) it is apparent that the CER/S1P rheostat is not regulated by RA as it does in cells with an intact RARα. In contrast, RA seems to activate, rather than downregulate, the SK signaling.

### SK1-S1P signaling: a candidate growth promoting mechanism of non-RAR-mediated RA action

**Figure 8:**
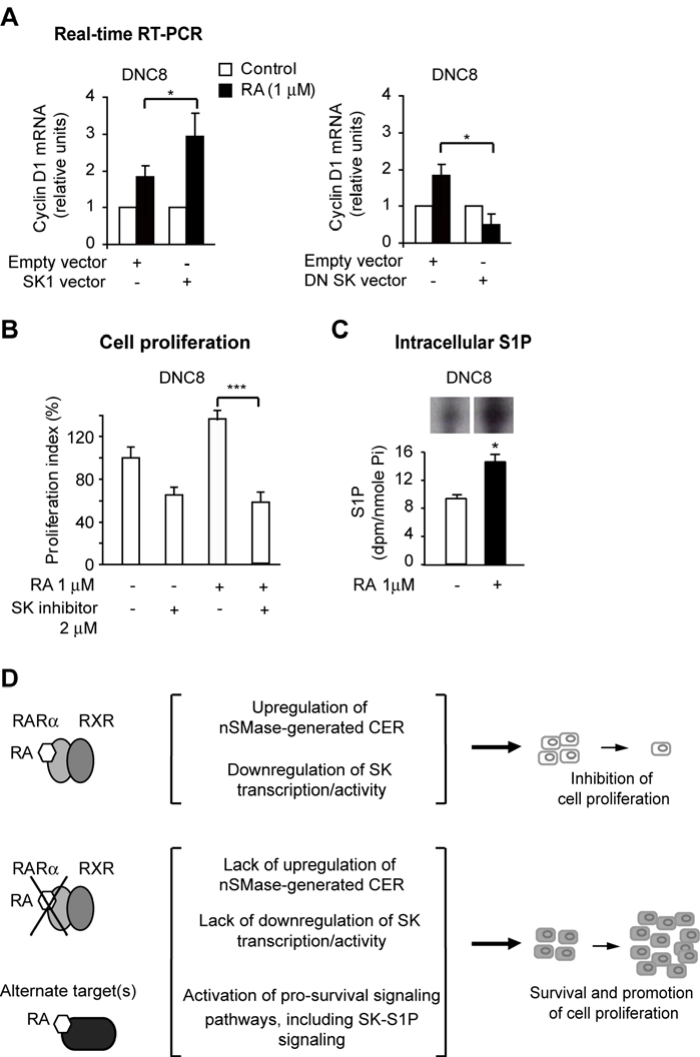
SK1-S1P signaling: a candidate growth promoting mechanism of non-RAR-mediated RA action. A) Transient exogenous expression of SK1 in DNC8 cells leads to upregulation of cyclin D1 transcription relative to cells expressing the cognate empty vector (left). Conversely, transient exogenous expression of a dominant negative SK mutant in DNC8 cells negatively affects RA-induced cyclin D1 transcription (right). B) RA-induced DNC8 proliferation is significantly decreased by treatment with a SK inhibitor. C) RA upregulates the S1P level (spots in the upper insert) in DNC8 cells. D) Scheme showing that RA action mediated through RARα results in upregulation of nSMase-generated CER sythesis, concomitant with downregulation of SK1 transcription/activity. These concerted antiproliferative metabolic changes concur to inhibit cell proliferation. Consequent to an impaired RA-RARα signaling, these concerted antiproliferative metabolic changes do not occur, thus cells survive in the presence of RA. Moreover, RA, through alternate, non-RAR (genomic or non-genomic) target(s), activates pro-survival signaling pathways, including the SK signaling pathway, thus leading to the expansion of the RA-resistant cell pool.

RA-induced cell survival and growth have been documented in different cells and tissues [33]–[35]. RA-induced proliferation in the absence of a functional RARα signaling is not breast cancer cell context-specific and can occur both in transformed and untransformed cells (unpublished observations). Apparently, a few non-RAR targets can mediate RA-action [34], [36], [37]. We gathered preliminary evidence that in DNC8 cells the RA non-RAR-mediated proliferation effect is due, at least in part, to activation of the SK1-S1P signaling pathway because of the following observations. First, in the presence of RA, *cyclin D1* transcription is upregulated in DNC8 cells transfected with wild-type SK1 (Fig. 8A, left). Second, exogenous expression of a dominant negative SK1 mutant (DNSK) in DNC8 cells significantly (p<0.05) reduced the level of *cyclin D1* transcription compared to the level of *cyclin D1* transcription in cells transfected with the cognate empty vector (Fig. 8A, right). Third, treatment with the specific SK inhibitor 2-(p-hydroxyanilino)-4-(p-chlorophenyl) thiazole, (2 µM, 72 h), led to significant inhibition (p<0.01) of DNC8 proliferation both in the absence, and presence of RA (Fig. 8B) as it did in MDA-MB-231 cells (Fig. 7). Finally, by labeling experiment with [3-^3^H] D-erythro-sphingosine, we observed a significant (p<0.05) increase of intracellular S1P in DNC8 cells in response to RA (1 µM, 72 h) (Fig. 8C).

We conclude that when RA is not channeled through RARα there is no longer concerted transcriptional upregulation of nSMase-mediated CER and transcriptional downregulation of SK1 activity. In contrast, RA, through alternate, non-RAR target(s), manages to activate the SK1-S1P signaling, thus promoting cell survival and growth (Fig. 8D).

## Discussion

In this study we provide mechanistic evidence that RA can act as a growth inhibitor or a growth promoter according to the functional status of RARα. Moreover, we provide evidence that in the presence of a functional RARα, RA inhibits cell growth by concertedly, and inversely, regulating the synthesis of two bioactive sphingolipids, CER and S1P. In contrast, we show that in the absence of RARα, RA, in a non-RAR-mediated fashion, promotes cell growth by activating the SK1-S1P-signaling. Specifically, we found that RA, when channeled through RARα in RA-sensitive cancer cells, concertedly upregulates on one hand nSMase, thus leading to accumulation of CER, the antiproliferative and proapoptotic sphingolipid, and on the other hand downregulates SK1, pivotal for the synthesis of the oncogenic S1P, the prosurvival sphingolipid. This regulation is lost in cells (T47D) where we stably inhibited RARα function with either a RARα antagonist, or a dominant negative RARα mutant and in cells (MDA-MB-231) that lack endogenous RARα function.

Lack of RA-RARα-mediated control of the sphingolipid rheostat explains why cells survive in the presence of RA. However, we found that cells not only survive, but actually grow more in the presence of RA. Thus, RA exerts a distinct effect that is non-RAR-mediated because lack of RARα determines the downregulation/silencing of the other two RARs, RARβ and RARγ (data not shown). The growth-promoting action of RA and its dietary precursors has puzzled investigators for many years. Beta-carotene was shown to increase, rather than reduce, the incidence of lung cancer [19], [22] and head and neck cancer [36]. Both retinol and RA were shown to promote tumor growth in transgenic models of both breast and colon cancer [18], [21], [35]. Here we show that chronic treatment with RA stimulates the growth of cells with an impaired RARα function not only *in vitro* but also *in vivo*. Thus, RA-induced expansion of cells may represent a discrete step of the tumor progression process once cells have lost RAR function. In the absence of functional RARs, RA apparently activates, through non-RAR targets, one or more pro-proliferative mechanisms. We provide evidence that one of these mechanisms is the SK1-S1P signaling. We do not know yet through which alternate, non-RAR target RA accomplishes to activate the SK1-S1P signaling. A potential non-RAR candidate target is protein kinase alpha (PKCα). PKCα can physically bind RA [37], can activate the SK signaling [38], and promote cell growth and tumor progression [39]. Another non-RAR candidate target is PPARβ/δ, an orphan nuclear receptor that binds with high affinity RA, recently implicated in RA-induced survival [35]. PKCα and PPARβ/δ are both expressed in our cell model with functionally disrupted RARα (data not shown).

Our study indicates that drugs such as fenretinide that can increase CER through pathways different from the RA-RARα-regulated nSMase pathway, or SK inhibitors can overcome the biological sequelae associated with the loss of RARα function and counteract RA-induced growth by targeting the sphingolipid rheostat. The identification of both non-RAR targets and mechanisms implicated in RA-mediated prosurvival/proliferation effects might bring us a step closer to the solution of the RA-paradox.

## Materials and Methods

### Cell cultures and biological assays

The T47D breast cancer cell line (ATCC, Manassas, VA) was cultured in DMEM medium (Invitrogen, Carlsbad, CA) plus 5% charcoal-dextran-stripped fetal bovine serum (Hyclone, Logan, UT). The T47D-derived clones, DNC8 and LXC5, carrying either a retroviral vector containing the human dominant-negative RARα403 mutant [40], or the cognate empty vector were developed as previously described [13]. The T47D-derived clone ER-C4 was developed by isolating and expanding single colonies that grew after treatment with RA 1 µM in combination with the RARα antagonist ER50891 as previously described [13]. Treatment with all-trans retinoic acid (RA) (Sigma, St Louis, MI), N-(4-hydroxyphenyl) retinamide (4-HPR) (Sigma), the RARα antagonists ER50891 (provided by Dr. Kouichi Kikuchi, Discovery Research Laboratories, Ibaraki, Japan) and RO415253 (provided by Dr. Salvatore Toma, Genoa, Italy), the sphingosine kinase inhibitor, 2-(p-hydroxyanilino)-4-(p-chlorophenyl) thiazole (Calbiochem, San Diego, CA), and the neutral sphingomyelinase inhibitor (GW4869) (Sigma) are described in detail in the Results. Cell proliferation was evaluated by either the 3-(4,5-dimethylthiazol-2-yl)-2,5-diphenyltetrazolium bromide (MTT) assay [41] or the Live/Dead Cell Viability assay (Invitrogen). For the colony formation assay, exponentially growing cells were seeded at 5×10^2^ cells/well in 6-well plates in triplicate and allowed to attach to the substrate. Cells were treated with or without RA 1 µM for 24 hours, and then the medium was replaced with drug-free medium and the cells grown for 14–21 days. Colonies were fixed with methanol and stained with Giemsa (Sigma). The total area of the colonies was assessed by using Image J (NIH). For flow cytometric cell cycle analysis, cells were trypsinized, washed with cold phosphate buffered saline (PBS), fixed in 70% ethanol at 4°C for 30 minutes, washed with cold 0.5% bovine serum albumin (BSA), resuspended in 1 ml Krishan Buffer containing 0.1% sodium citrate, 0.02 mg/ml RNase A (Qiagen, Valencia, CA), 0.20% NP40 (Sigma), 0.05 mg/ml propidium iodide (Sigma), kept at 4°C for 30 minutes, and analyzed with a Fluorescence-Activated Cell Sorter (FACS) (Becton Dickinson Biosciences, San Jose, CA) equipped with Cellquest software. The data were analyzed with ModFit LT software (Verity Software House, Inc. Topsham, ME). Transient transfection was performed by transfecting 5×10^5^ cells attached to the plastic substrate with either a vector carrying the dominant negative SK1 (pcDNA3-hSKG82D) (provided by Dr. Stuart M. Pitson, Hanson Institute, Adelaide, Australia) [42], or the cognate empty vector (pcDNA3, Invitrogen), using LipofectAmine Plus (Invitrogen).

### Animal studies and tumor analysis

Female athymic NCr-nu/nu mice (6–8 weeks old) were bought from NCI-Frederick Animal Production Program (NCI, Frederick, MD). All mice were kept in a temperature-controlled room on a 12/12-h light/dark schedule, with food and water *ad libitum*. Mice were estrogenized by intramuscular injection of Depo-estradiol (Florida Infusion Co, Palm Harbor, FL) at 1.5 mg/kg body weight. Two days after, mice were subcutaneously inoculated in the flank region (bilaterally) with either 5×10^6^ DNC8 cells (16 mice) or 5×10^6^ LXC5 cells (16 mice) in 0.2 ml of a mixture of serum-free DMEM (Invitrogen) and Matrigel (BD Biosciences, Bedford, MA) (1∶1, by vol). Mice inoculated with either LXC5 or DNC8 cells were randomly divided into two groups of 8 mice each. When mice developed palpable tumors (approximate tumor size of 20 mm^3^), they were treated with either the vehicle, dimethylsufoxide (DMSO), or RA (2.5 mg/kg body weight) by intraperitoneal (i.p.) injection five times a week, up to six weeks. Tumors size was measured with a digital caliper twice a week, and tumor volumes were calculated as described [43]. Mice were monitored and weighed weekly. At the end of the sixth week, mice were euthanized. Data were analyzed by one-way ANOVA, followed by multiple comparison tests (STATISTICA program, Tulsa, OK, USA). All statistical tests were two-sided. The level of significance was set at p<0.05.

Three tumors (right side) randomly selected from each group of mice were removed and cut in half. One half was snap-frozen in liquid nitrogen and used for evaluating *cyclin D1* transcription by quantitative real time RT-PCR. The other half was fixed in 10% neutral-buffered formalin and used for immunohistochemical analysis of the Ki67, a parameter of cell proliferation. Fixed tissues embedded in paraffin were sectioned. 5 µm-sections were reacted with either a rabbit anti-Ki67 human antibody (Dako, Carpinteria, CA) or horse serum as a control, followed by a biotinylated horse anti-rabbit antibody (BioGenex, San Ramon, CA), and visualized by using streptavidin horseradish peroxidase/diaminobenzidine. Sections were counterstained with hematoxylin and mounted. Ki67-positive cells were quantified as described [44]. The differences between two-pair samples were analyzed by the two-tailed Student's *t* test.

### Quantitative real-time RT-PCR

Total RNA isolated by Trizol (Invitrogen) followed by DNase I (Qiagen) treatment, and retrotranscribed into cDNA with a SuperScript First-Strand Synthesis System (Invitrogen) was amplified with iQ SYBR Green Supermix kit (BioRad, Hercules, CA) in combination with specific primers using a MyiQ Real-Time PCR Detection System (BioRad). The primer sequences were as follows: *cyclin D1* (sense: 5′-CTG TGC TGC GAA GTG GAA ACC AT-3′; antisense: 5′-TGG AGT TGT CGG TGT AGA TGC ACA-3′), *nSMase* (sense: 5′-CAA CAA GTG TAA CGA CGA TGC C-3′; antisense: 5′-CGA TTC TTT GGT CCT GAG GTG T-3′), *SK1* (sense: 5′-CTG GCA GCT TCC TTG AAC CAT-3′; antisense: 5′-TGT GCA GAG ACA GCA GGT TCA-3′); *LCB1* (sense, 5′-TTA ACT CAG GCG CGC TAC TTG-3′ and antisense, 5′-TGT TGT TCC ACC GTG ACC A-3′); *LCB2* (sense, 5′-GCC ACC CCA ATT ATT GAG TCC-3′ and antisense, 5′-TGC AAT AGG TCC CCA ACT TCA-3′); *SK2* (sense, 5′-CCA GTG TTG GAG AGC TGA AGG T-3′ and antisense, 5′-GTC CAT TCA TCT GCT GGT CCT C-3′); *aSMase* (sense, 5′-TGG CTC TAT GAA GCG ATG GC-3′ and antisense, 5′-TTG AGA GAG ATG AGG CGG AGA C-3′); *S1P phosphatase* (sense, 5′-TGA GTA CAG CAT GCC CTC CA-3′ and antisense, 5′-GGC AAA CTA GAG AAC ACC AGC A-3′); *S1P lyase* (sense, 5′-GAG CAC CCA TTT GAT TTC CG-3′ and antisense, 5′-CAC CAA TGA TGA GCC TTT TGG-3′); *GAPDH* (sense: 5′-GAA GGT GAA GGT CGG AGT C-3′; antisense: 5′-GAA GAT GGT GAT GGG ATT TC-3′). The level of the different transcripts was normalized to the level of the *GAPDH* transcript, and quantified by the threshold cycle Ct method.

### Diacylglycerolkinase (DGK) assay

Lipids were extracted according to the method of Bligh and Dyer [45]. CER was quantitated with the DGK assay [46]. Briefly, 30 nmol of lipids, quantitated as inorganic phosphate [47], were incubated in the presence of 20 µl β-octylglucoside/dioleoylphosphatidylglycerol micelles [48], 2 mM dithiothreitol, 6 µg of Escherichia coli DGK (Calbiochem), 1 mM ATP, 1.3 µCi of [γ^32^-P] ATP (3 Ci/µmol) (Perkin Elmer Life Sciences Inc., Boston, MA) in a final volume of 100 µl at 25°C for 45 minutes. Radioactive lipids were separated, along with reference lipid standards (Avanti Polar Lipids, Alabaster, AL), by thin layer chromatography (TLC), with chloroform/acetone/methanol/acetic acid/water (10/4/3/2/1, by vol). Radioactive CER phosphate spots were visualized by autoradiography, scraped, and counted by liquid scintillation.

### Quantitation of *de-novo* generated ceramide by [^3^H] palmitate labeling

6×10^5^ cells were labeled with 4 µCi of [9,10(n)-^3^H] palmitate (55 Ci/mmol) (Amersham Biosciences Italy, Italy) in the presence of either RA (1 µM) or ethanol (vehicle) at 37°C for 10 h. Lipids, extracted as described previously, were subjected to mild alkaline hydrolysis (0.1 N NaOH in methanol, at 55°C for 1 h), 48 h-dialysis against distilled water, lyophilized, resuspended in 50 µl chloroform/methanol (2/1, by vol) and separated along with reference standards by TLC using chloroform/methanol/2N NH_4_OH (40/7.5/1, by vol). Radioactive CER spots were visualized by fluorography, scraped, and counted by liquid scintillation.

### Quantitation of SMase-generated ceramide by [^3^H] sphingosine labeling

6×10^5^ cells were labeled with 0.4 µCi [3-^3^H]D-erythro-sphingosine (23 Ci/mmoles) (Perkin Elmer) at 37°C for 72 h, before adding RA (1 µM) or ethanol (vehicle) for additional 72 h. Lipids were extracted as described above. Radioactive lipids and reference standards were resolved by TLC using either chloroform/methanol/2N NH_4_OH (40/7.5/1, by vol) for CER separation or chloroform/methanol/formic acid/water (65/25/8.9/1.1, by vol) for sphingomyelin separation. Radioactive CER and sphingomyelin spots, visualized by fluorography and recognized by comparison with reference standards, were scraped and counted by liquid scintillation.

### Acid sphingomyelinase (aSMase) activity assay

aSMase activity was determined essentially as described [49]. Briefly, cells were lysed by three freeze-thawing cycles in 200 µl of a lysis buffer containing 50 mM Tris-HCl (pH 7.4), 1 mM EDTA, 0.1% Triton X-100, 5 mM dithiothreitol, 1 mM phenylmethylsulfonyl fluoride, and 2 µl of a protease inhibitor cocktail (Sigma). The lysate was centrifuged at 1000×g for 15 min. The supernatant was collected, and protein content determined by Comassie Plus assay (Pierce Biotechnology, Inc, Rockford, IL). The protein concentration was adjusted at 1 µg/µl with lysis buffer. 50 µg proteins were added to 50 µl solution, which was previously sonicated for 30 sec, containing 200 mM sodium acetate, pH 5.0, 0.1% Triton X-100, 0.5 µCi [N-methyl-^14^C] sphingomyelin (54.0 mCi/mmol) (Amersham Biosciences) and 100 µM sphingomyelin (Sigma) and incubated at 37°C for 60 min. The reaction was stopped by adding 1.5 ml chloroform/methanol (2/1, by vol) and 0.2 ml distilled water. Phases were separated by centrifugation at 2000×g for 5 min. Upper aqueous phase aliquots were counted by liquid scintillation.

### Neutral sphingomyelinase (nSMase) activity assay

nSMase activity was determined essentially as described [49]. Briefly, 9×10^6^ cells were lysed by three freeze-thawing cycles in 200 µl of a lysis buffer containing 50 mM Tris-HCl (pH 7.4), 1 mM EDTA, 0.1% Triton X-100, 5 mM dithiothreitol, 1 mM phenylmethylsulfonyl fluoride, and 2 µl of a protease inhibitor cocktail (Sigma). The cell lysate was centrifuged at 1000×g for 15 min. The supernatant was collected for protein content determination by the Comassie Plus assay (Pierce Biotechnology, Inc.). The protein concentration was adjusted to 1 µg/µl with the lysis buffer. 50 µg proteins were added to 50 µl of a solution, which was previously sonicated for 30 sec, containing 100 mM Tris-HCl (pH 7.4), 10 mM MgCl_2_, 0.2% Triton X-100, 10 mM dithiothreitol, 0.5 µCi [N-methyl-^14^C] sphingomyelin (54 mCi/mmol) (Amersham Biosciences, Buckingamshire, UK), and 100 µM sphingomyelin (Sigma), and incubated for 60 min at 37°C. The reaction was stopped by adding 1.5 ml chloroform/methanol (2/1, by vol) and 0.2 ml distilled water. Phases were separated by centrifugation at 2000×g for 5 min. Upper aqueous phase aliquots were counted by liquid scintillation.

### Serine palmitoyltransferase (SPT) activity assay

The activity of SPT was determined essentially as described [50]. Briefly, 1×10^7^cells were lysed by three freeze-thawing cycles in 300 µl of a lysis buffer containing 25 mM Hepes (pH 7.4), 5 mM EGTA, 50 mM NaF, 3 µl of a protease inhibitor cocktail (Sigma). The cell lysate was centrifuged at 1000×g for 15 min. The supernatant was collected and protein content determined by Comassie Plus assay (Pierce Biotechnology, Inc.). The protein concentration was adjusted to 5 µg/µl with lysis buffer. 200 µg proteins were added to 160 µl solution containing 100 mM Hepes (pH 8.3), 2.5 mM EDTA, pH 7.4, 50 µM pyridoxalphosphate, 5 mM dithiothreitol, 1 mM L-serine (200 µl final volume). After a pre-incubation at 37°C for 5 min, 1 µCi L-[^3^H(G)]serine (26.0 Ci/mmol) (Perkin Elmer) and 20 µl 2 mM palmitoyl CoA were added. Incubation was allowed to proceed for 20 min at 37°C and stopped by adding 1.5 ml of chloroform/methanol (1/2, by vol), 25 µg D-erythro-sphingosine (Avanti Polar), 1.5 ml chloroform and 2 ml 0.5 N NH_4_OH. Phases were separated by centrifugation at 2000×g for 5 min. The lower organic phase was washed twice with 2 ml distilled water. Aliquots were dried and counted by liquid scintillation.

### Sphingosine kinase (SK) activity assay

SK activity was determined essentially as described [51]. Briefly, 9×10^6^ cells were lysed by three freeze-thawing cycles in 200 µl of a lysis buffer containing 20 mM Tris-HCl (pH 7.4), 10% glycerol, 1 mM β-mercaptoethanol, 1 mM EDTA, 1 mM sodium orthovanadate, 15 mM NaF, 40 mM β-glycerophosphate, 0.5 mM deoxypiridoxine, 0.1% Triton X-100, 2 µl of a protease inhibitor cocktail (Sigma). The cell lysate was centrifuged at 13,000×g for 30 min. The supernatant was collected for protein content determination by the Comassie Plus assay (Pierce Biotechnology, Inc.). The protein concentration was adjusted to 1 µg/µl by adding lysis buffer. 100 µg (100 µl) protein were added to 10 µl of 1 mM D-erythro-sphingosine (Avanti Polar) dissolved in 0.1% Triton X-100 and 10 µl of a solution containing 10 µCi [γ^32^P] ATP (3 Ci/µmol) (Perkin Elmer), 20 mM MgCl_2_, 2 mM ATP, and incubated at 37°C for 30 min. The reaction was stopped by adding 20 µl 1 N HCl and 0.8 ml chloroform/methanol/37% HCl (100/200/1, by vol ) followed after 10 min by the addition of 250 µl chloroform and 250 µl 2 M KCl. Phases were separated by centrifugation at 2000×g for 5 min. The lower organic phase was collected, dried and redissolved in 50 µl of chloroform/methanol/37% HCl (100/200/0.2, by vol). Radioactive lipids were resolved by TLC using n-butanol/acetic acid/water (3/1/1, by vol). Labeled S1P, visualized by autoradiography and recognized by comparison with a reference standard, was scraped and counted by liquid scintillation.

### Intracellular S1P assay

Intracellular S1P was determined essentially as described [52]. 6×10^5^ cells were labeled with 0.8 µCi of [3-^3^H]D-erythro-sphingosine (23 Ci/mmoles) (Perkin Elmer) and 50 pmoles of sphingosine (Avanti Polar) in 100 µl DMEM, for 2 h at 37°C. Lipids were extracted by addition of 1.9 ml chloroform/methanol/37% HCl (100/200/1, by vol), 625 µl chloroform and 625 µl 2 M KCl. Phases were separated by centrifugation at 2000×g for 5 min. The lower organic phase was collected, dried, and dissolved in 30 µl of chloroform/methanol (2/1, by vol). Radioactive lipids were resolved by TLC using n-butanol/acetic acid/water (3/1/1, by vol). Labeled S1P, visualized by fluorography and recognized by comparison with a reference standard, was scraped and counted by liquid scintillation.

### Statistical analysis

Unless specifically stated, data represent the mean of three independent experiments±standard deviation (SD). The significance of differences between groups was obtained by the Student's t-test. In the Figures one asterisk correspond to p<0.05, two asterisks to p<0.01 and three asterisks to p<0.001.
